# Memantine in patients with Alzheimer's disease receiving donepezil: new analyses of efficacy and safety for combination therapy

**DOI:** 10.1186/alzrt160

**Published:** 2013-01-21

**Authors:** Alireza Atri, José L Molinuevo, Ole Lemming, Yvonne Wirth, Irena Pulte, David Wilkinson

**Affiliations:** 1 Department of Neurology, Massachusetts General Hospital, Memory Disorders Unit, 15 Parkman Street, WACC 715, Boston, MA 02114, USA; 2 Geriatric Research Education and Clinical Center, Edith Nourse Rogers Memorial Veterans Hospital, 200 Springs Road, Bedford, MA 01730, USA; 3 Harvard Medical School, 25 Shattuck Street, Boston, MA 02115, USA; 4 Alzheimer's Disease and Other Cognitive Disorders Unit, Neurology Service, Hospital Clínic i Universitari, Villarroel 170, Barcelona, 08036, Spain; 5 Biostatistics Department, H. Lundbeck A/S, Ottiliavej 9, DK-2500 Valby, Copenhagen, Denmark; 6 Wirth Consulting, Gauss Strasse 42, Stuttgart D-70193, Germany; 7 Global Clinical R & D CNS, Merz Pharmaceuticals GmbH, Eckenheimer Landstraße 100, Frankfurt am Main, 60318, Germany; 8 Memory Assessment & Research Centre, Tom Rudd Unit, Moorgreen Hospital, Botley Road, West End, Southampton, SO30 3JB, UK

## Abstract

**Introduction:**

Memantine and cholinesterase inhibitors potentially offer additional benefits in Alzheimer's disease (AD) when used together. This study assessed the efficacy and safety of combination treatment with memantine added to stable donepezil in patients with moderate to severe AD, and in a subset with moderate AD.

**Methods:**

Post hoc meta-analyses of data combined from two 24-week, randomised, double-blind, placebo-controlled trials of memantine 20 mg/day versus placebo, added to a stable cholinesterase inhibitor, were conducted. Data were included for all patients receiving donepezil 10 mg/day with Mini-Mental State Examination (MMSE) scores < 20 (*n *= 510). Efficacy was assessed using measures of cognition, function, and global status. Furthermore, marked clinical worsening, defined as concurrent deterioration from baseline in the three main efficacy domains, and safety, measured by treatment-emergent adverse events, were assessed. Analyses were performed for patients with moderate to severe AD (MMSE 5-19; MOD-SEV subgroup), and also for patients with moderate AD (MMSE 10-19; MOD subgroup; *n *= 367).

**Results:**

At week 24, in the MOD-SEV subgroup, patients receiving memantine added to donepezil significantly outperformed those receiving placebo added to donepezil in measures of cognition (*P *< 0.0001), function (*P *= 0.02), and global status (*P *= 0.010), with standardised mean differences (SMDs) of 0.36, 0.21, and 0.23, respectively (all last observation carried forward). Similarly, in the MOD subgroup, significant benefits were observed for cognition (*P *= 0.008), function (*P *= 0.04) and global status (*P *= 0.008), with SMDs of 0.28, 0.21, and 0.28, respectively. Significantly fewer patients receiving memantine added to donepezil showed marked clinical worsening than those receiving placebo added to donepezil, in both subgroups (MOD-SEV: 8.7% versus 20.4%, *P *= 0.0002; MOD: 5.9% versus 15.0%, *P *= 0.006). The incidence of adverse events was similar between treatment groups.

**Conclusions:**

These results support and extend previous evidence that combination treatment with memantine added to stable donepezil in patients with moderate AD, and in those with moderate to severe AD, is associated with significant benefits in reducing 24-week decline in cognition, function and global status. Combination treatment produces substantially reduced rates of marked clinical worsening, has good safety and tolerability, and generates effect sizes that are both statistically significant and clinically meaningful.

## Introduction

Alzheimer's disease (AD) is a progressive neurodegenerative disorder in which patients typically lose cognitive faculties, struggle to carry out activities of daily living (ADLs), and experience behavioural and neuropsychiatric problems. At present, AD cannot be cured, any improvements produced by pharmacotherapy are often temporary, and no treatments have been demonstrated to be disease-modifying. Consequently, alleviating symptoms, and delaying or reducing clinical worsening (that is, symptom progression), without modifying the underlying pathophysiology, are realistic and meaningful treatment goals [1] that can be termed disease-course-modifying effects [2]. Achieving these goals allows patients to spend more time in the milder, more functional, stages of AD than they would without treatment [1].

Memantine, an uncompetitive antagonist of *N*-methyl-D-aspartate (NMDA) glutamate receptors, is approved in the EU and US for the treatment of patients with moderate to severe AD (Mini-Mental State Examination [MMSE] [3] score < 20). Donepezil, a cholinesterase inhibitor (ChEI), is approved for the treatment of mild to moderate AD in the EU, and for mild, moderate, and severe AD in the US and some other countries. As monotherapy, both memantine and donepezil have demonstrated efficacy for treating the symptoms of AD within their respective approved indications [4-12]. In addition, the incidence of clinical worsening, as defined by concurrent deterioration in three domains (cognitive, functional, and global) over time, is reduced by memantine treatment in patients with moderate to severe AD [6], and by donepezil treatment in patients with mild to moderate AD [13].

Since memantine and donepezil have different and complementary mechanisms of action, together they potentially offer additional benefits to the patient [14]. Pharmacokinetic and pharmacodynamic data in healthy volunteers provided initial evidence that memantine and donepezil may be safely used in combination [15]. The addition of memantine to stable ChEI therapy has also been associated with a good safety profile in patients with AD [16,17].

Two 24-week, randomised, double-blind, placebo-controlled trials (RCTs) have investigated the efficacy and safety of memantine 20 mg/day in combination with a ChEI. The first, MEM-MD-02, assessed the efficacy of administration of memantine (10 mg twice daily) versus placebo in patients with moderate to severe AD (MMSE 5-14; *n *= 404) receiving stable donepezil therapy [16]. Relative to placebo, memantine produced significant benefits in all four key symptom domains of AD, namely, cognition, function, behaviour, and global status [16]. The second RCT, MEM-MD-12, assessed the efficacy of administration of memantine (20 mg once daily) versus placebo in patients with mild to moderate AD (MMSE 10-22; *n *= 433) taking a stable dose of any approved ChEI therapy [17]. In this trial, the only potential signal of benefit for memantine treatment over placebo (effect size estimate 0.118 in favour of memantine; *P *= 0.184) was observed for the cognitive measure (AD Assessment Scale-cognitive subscale, ADAS-Cog), but the trial was not adequately powered to detect with statistical significance, an effect size smaller than 0.325 [17]. In both studies, combination therapy with memantine added to a ChEI was well-tolerated [16,17].

In addition to the lack of power to detect effect sizes smaller than 0.325, two possible explanations were provided by Porsteinsson and colleagues for the discrepancy in findings between MEM-MD-02 and MEM-MD-12: the difference in baseline disease severity, and the difference in permitted ChEIs [17]. In the present study, data from both RCTs are combined, and the hypothesis that low power and baseline heterogeneities caused the divergent results between MEM-MD-02 and MEM-MD-12, and potentially obscured significant memantine treatment-related benefits in patients with moderate AD, is tested. In a post hoc meta-analysis and subgroup analysis approach, these data are used to assess the efficacy of memantine 20 mg/day versus placebo in patients receiving stable doses of donepezil (10 mg/day) in two subgroups: moderate to severe AD (MMSE 5 to 19), and moderate AD (MMSE 10 to 19).

The rationale for choosing these patient subgroups (subpopulations) were that they represent the current approved indication of memantine in the EU (moderate to severe AD), and the overlap of the approved memantine and donepezil indications in the EU (moderate AD). As is commonly done in clinical trials, the MMSE was used as a subpopulation staging surrogate measure to delineate mild (MMSE ≥ 20) from moderate (MMSE 10 to 19) and severe (MMSE < 10) stages of AD. Finally, since donepezil was the most commonly used ChEI in these trials, ChEIs other than donepezil were excluded, and analysis was restricted to patients receiving 10 mg/day of donepezil to minimise heterogeneity and any potential effects of underdosing.

Therefore, the analyses in this study of patients with AD with MMSE < 20 taking stable donepezil 10 mg/day consist of: 1) meta-analyses to compare the efficacy of memantine versus placebo across individual domains of AD; 2) pooled analyses to compare the efficacy of memantine versus placebo in reducing the occurrence of marked clinical worsening, and 3) pooled analyses to assess the tolerability profile of memantine versus placebo.

## Methods

### Study design and patients

**Figure 1:**
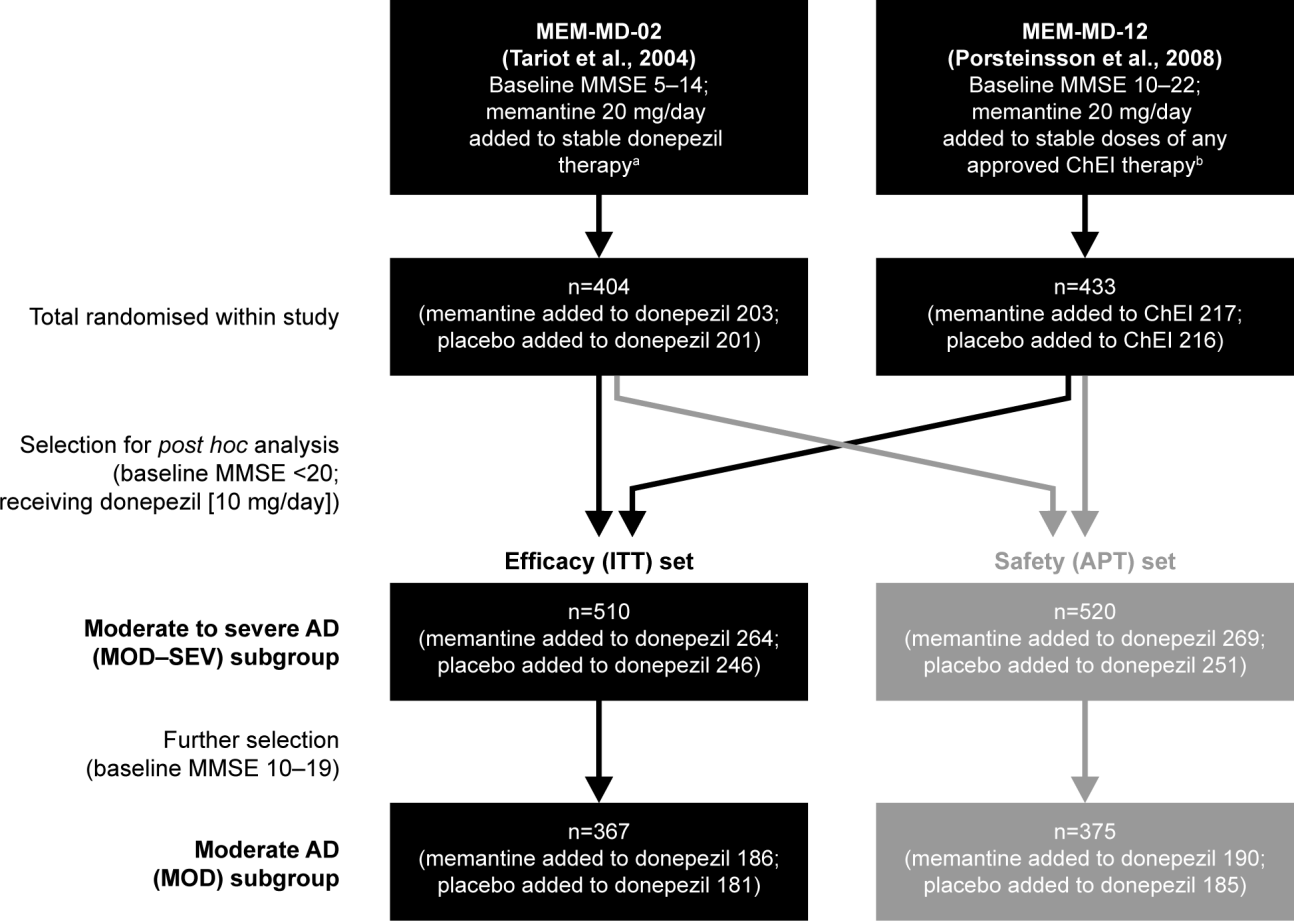
**Patient flow**. ^a^5 to 10 mg/day for ≥ 3 months. ^b^5 or 10 mg/day donepezil; 6, 9 or 12 mg/day rivastigmine; 16 or 24 mg/day galantamine for ≥ 3 months. MMSE, Mini-Mental State Examination; ChEI, cholinesterase inhibitor; ITT, intention-to-treat; APT, all-patients-treated; AD, Alzheimer's disease.

Figure 1 depicts data flow for study inclusion and subgroup analysis. Studies were selected for inclusion if they fulfilled the criteria of: phase III RCTs of patients with a diagnosis of AD and treated with memantine 20 mg/day added to stable ChEI; a double-blind observation period of at least 24 weeks, and a majority of patients receiving stable treatment with donepezil. Two studies met the inclusion criteria (MEM-MD-02 and MEM-MD-12), both of which were performed in multiple centres in the US [16,17]. Study approval was granted by the local Institutional Review Board (IRB) at each trial site, and written informed consent was obtained from each study participant if possible, and either the caregiver or a legally acceptable representative (if different from the caregiver) before initiation of study-specific procedures according to IRB protocols [16,17].

Full details of individual study design and patient inclusion criteria for MEM-MD-02 and MEM-MD-12 have been presented previously [16,17]. In summary, the patient inclusion criteria were similar: 50 years of age or older; diagnosis of probable AD according to the NINCDS-ADRDA criteria; a brain magnetic resonance imaging or computed tomographic scan within 12 months consistent with a diagnosis of probable AD; and treatment with a ChEI for at least 6 months with a stable dosing regimen for at least 3 months. The individual clinical study inclusion criteria differed in the required baseline MMSE score (see Figure 1) and the allowed ChEI (only donepezil use in MEM-MD-02; any ChEI in MEM-MD-12). In both studies, patients treated with memantine received a fixed total dose of 20 mg/day.

Selection was restricted to patients receiving stable treatment with donepezil 10 mg/day. Two subgroups of patients were analysed: the MOD to SEV subgroup of patients with moderate to severe AD (MMSE < 20, range 5 to 19), conforming to the approved indication of memantine in the EU, and the MOD subgroup of patients with moderate AD (MMSE 10 to 19), conforming to the overlap of the approved memantine and donepezil indications in the EU.

### Trial registration

The data were obtained from the sponsors of the original trials; trial registration was not relevant to these two studies since both studies were completed before July 1, 2005; MEM-MD-02 was completed by June 2002, and MEM-MD-12 was completed by March 2003 [16,17].

### Efficacy measures

Cognition was assessed using the Severe Impairment Battery (SIB) [18-20] in study MEM-MD-02 (patients with moderate to severe AD) and the ADAS-Cog [21] in study MEM-MD-12 (patients with mild to moderate AD). In both studies, function was assessed using the AD Cooperative Study - Activities of Daily Living scale (ADCS-ADL) [22,23]. The 23-item version (ADCS-ADL_23_) was used in MEM-MD-12, and the 19-item version (ADCS-ADL_19_), developed specifically for patients with moderate to severe AD [23], was used in MEM-MD-02. Global status was assessed using the Clinician's Interview-Based Impression of Change Plus Caregiver Input (CIBIC-Plus) [24,25].

An individual domains meta-analysis was performed for each subgroup. This required data from the different cognitive and functional rating scales within the same domain to be combined across the selected trials. Consequently, the outcome measures for this meta-analysis were change from baseline to endpoint (week 24) in cognition (SIB/ADAS-Cog score), function (ADCS-ADL_19_/ADCS-ADL_23 _score), and global status (CIBIC-Plus score).

Data from both studies were pooled in clinical worsening analyses, a form of responder analysis in which response is defined not by improvement but by worsening [6]. The criteria used to define clinical worsening were based on concurrent worsening in the cognitive, functional and global domains from baseline to endpoint (week 24) [6]. Marked clinical worsening was defined as a decline of ≥ 4 points on ADAS-Cog or ≥ 5 points on SIB, plus any decline on ADCS-ADL_19_/ADCS-ADL_23 _and CIBIC-Plus [6]. This definition is intended to represent the average natural cognitive decline observed in patients with moderate to severe AD over 6 months, and can be considered as clinically significant cognitive worsening [6].

Finally, safety and tolerability were assessed in a pooled analysis of adverse events (AEs), including both the total incidence of AEs, and the AEs with an incidence ≥ 5% in either treatment group.

### Statistical analysis

All analyses were performed using SAS^® ^9.2 and RevMan 5 software. Efficacy was analysed in the intention-to-treat (ITT) set, defined as all patients who were randomised to, and received at least one dose of, either placebo or memantine, and who completed at least one post-baseline assessment in the cognitive (SIB/ADAS-Cog) or functional (ADCS-ADL_19/23_) domains. Analyses were conducted at week 24 using the last observation carried forward (LOCF) approach for missing data, and also for observed cases (OC).

For the individual studies in the meta-analyses, standardised effect sizes for each outcome measure were calculated as the standardised mean difference (SMD) of the change from baseline to endpoint. The overall standardised effect size for each outcome measure was calculated using the inverse-variance method. Per convention, we use Cohen's guidelines to serve as operational definitions to qualitatively interpret the magnitude of effect sizes as follows: 0.2 is small, 0.5 is medium, and 0.8 is large [26]. Effect sizes of magnitude 0.2 or larger are considered clinically significant in the context of general medical therapeutics [26], and are also clinically noticeable in the context of AD therapeutics [27]. The meta-analyses were conducted using the fixed-effect model. Overall effect was tested using the *Z*-statistic. Statistical testing for heterogeneity was based on chi squared tests and the *I*-squared summary statistic; heterogeneity between studies was considered for *P*-values < 0.10, or *I*-squared > 50%. The pooled analyses of clinical worsening included patients in the ITT set who had an assessment on all three efficacy scales.

Safety was assessed in the all-patients-treated (APT) set, defined as all patients who were randomised to, and received at least one dose of, either placebo or memantine. Significance was calculated using Fisher's exact test; *P*-values < 0.05 were considered statistically significant.

## Results

### Study population

A total of 510 patients with moderate to severe AD (MOD-SEV subgroup; MMSE 5 to 19) (339 from MEM-MD-02, and 171 from MEM-MD-12; 264 receiving memantine added to donepezil, and 246 receiving placebo added to donepezil) were included in the ITT set. Of these, 367 patients (186 receiving memantine added to donepezil, and 181 receiving placebo added to donepezil) were part of the MOD subgroup (MMSE 10 to 19) (Figure 1). As expected, other than baseline MMSE score, there were no clinically relevant differences between treatment groups in terms of baseline demographics (Table 1).

**Table 1:** Baseline patient demographics and MMSE scores (ITT set)

	MOD-SEV subgroup^a^		MOD subgroup^b^	
	
Characteristic	Memantine added to donepezil (*n *= 264)	Placebo added to donepezil (*n *= 246)	Memantine added to donepezil (*n *= 186)	Placebo added to donepezil (*n *= 181)
Female	155 (58.7)	151 (61.4)	114 (61.3)	110 (60.8)
Age, mean (SD) years	75.1 (8.5)	75.8 (8.5)	75.9 (8.4)	76.4 (8.2)
Caucasian	236 (89.4)	230 (93.5)	166 (89.2)	171 (94.5)
Black	10 (3.8)	6 (2.4)	5 (2.7)	4 (2.2)
Asian	3 (1.1)	1 (0.4)	3 (1.6)	1 (0.6)
Other	15 (5.7)	9 (3.7)	12 (6.5)	5 (2.8)
MMSE score, mean (SD)	11.9 (3.9)	11.7 (3.7)	13.9 (2.5)	13.4 (2.6)

Data are number (%) unless otherwise specified. ^a^Moderate to severe Alzheimer's disease (AD) (MMSE 5 to 19 at baseline), receiving donepezil (10 mg/day). ^b^Moderate AD (MMSE 10 to 19 at baseline), receiving donepezil (10 mg/day). MMSE, Mini-Mental State Examination; ITT, intention-to-treat.

### Excluded patient population characteristics

Table 2 shows the baseline characteristics for the 327 patients (mean baseline MMSE 18.0) originally enrolled in MEM-MD-02 (65 of 404 patients) or MEM-MD-12 (262 of 433 patients) who did not meet inclusion criteria for this study and were excluded from the efficacy analysis. Of the excluded patients who were part of the ITT set, 130 (all from MEM-MD-12; 90 receiving donepezil and 40 receiving a ChEI other than donepezil) met exclusion criteria for mild-stage AD (a baseline MMSE score of ≥ 20); there were no significant differences in baseline MMSE between patients randomised to memantine (*n *= 63; MMSE = 21.1) or placebo (*n *= 67; MMSE = 21.0). A further 100 patients from the ITT set (all from MEM-MD-12) met baseline MMSE criteria for moderate AD (MMSE 10 to 19) but were excluded for receiving a ChEI other than donepezil; these patients also had no significant differences in baseline MMSE between those randomised to memantine (*n *= 47; MMSE 14.1) or placebo (*n *= 53; MMSE 15.0). Finally, 81 patients from the ITT set (56 from MEM-MD-02 and 25 from MEM-MD-12) were excluded for taking a dose of donepezil less than 10 mg/day.

**Table 2:** Baseline characteristics of patients excluded due to any reason^a^

	All excluded patients	
	
Characteristic	Memantine (*n *= 156)	Placebo (*n *= 171)
Female	91 (58.3)	92 (53.8)
Age, mean (SD) years	75.4 (7.3)	75.7 (8.7)
Caucasian	147 (94.2)	162 (94.7)
Black	4 (2.6)	3 (1.8)
Asian	0 (0.0)	1 (0.6)
Other	5 (3.2)	5 (2.9)
MMSE score, mean (SD)	17.9 (4.0)	18.0 (3.6)

Data are number (%) unless otherwise specified. ^a^Exclusion criteria were mild Alzheimer's disease (MMSE ≥ 20 at baseline), receiving a cholinesterase inhibitor other than donepezil, or receiving donepezil at a dose < 10 mg/day. MMSE, Mini-Mental State Examination.

### Efficacy in individual domains of AD (meta-analyses)

**Figure 2:**
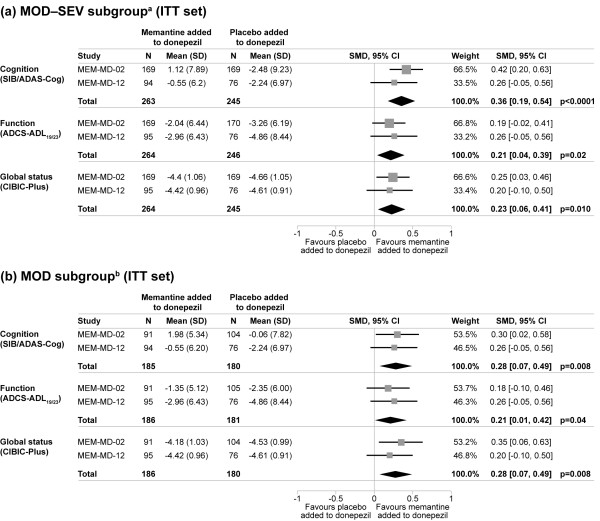
**Meta-analyses of change from baseline to endpoint in individual domains of Alzheimer's disease (LOCF analysis)**. ^a^Moderate to severe (AD) (MMSE 5 to 19 at baseline), receiving donepezil (10 mg/day). ^b^Moderate AD (MMSE 10 to 19 at baseline), receiving donepezil (10 mg/day). AD, Alzheimer's disease; ITT, intention-to-treat; LOCF, last observation carried forward; SMD, standardised mean difference; SIB, Severe Impairment Battery; ADAS-Cog, AD Assessment Scale-cognitive subscale; ADCS-ADL_19/23_, 19-/23-item AD Cooperative Study-Activities of Daily Living scale; CIBIC-Plus, Clinician's Interview-Based Impression of Change Plus Caregiver Input.

After 24 weeks of treatment, patients in the MOD-SEV subgroup receiving memantine added to donepezil showed significantly better efficacy across all examined domains of cognition, function, and global status than patients treated with placebo added to donepezil. The overall standardised effect sizes for memantine versus placebo were: 0.36 (*P *< 0.0001) for cognition, 0.21 (*P *= 0.02) for function, and 0.23 (*P *= 0.010) for global status (all LOCF; see Figure 2a). OC analyses produced similar results for statistical significance and standardised effect sizes. There was no sign of heterogeneity in either LOCF or OC analyses.

Treatment with memantine added to donepezil was also associated with significant clinical benefits in the MOD subgroup. The overall standardised effect sizes for memantine versus placebo were: 0.28 (*P *= 0.008) for cognition, 0.21 (*P *= 0.04) for function, and 0.28 (*P *= 0.008) for global status (all LOCF; see Figure 2b). In OC analyses, memantine treatment was associated with statistical significance only for the global status measure, but similar overall standardised effect sizes were observed. There was no sign of heterogeneity in either LOCF or OC analyses.

### Efficacy in reducing the occurrence of marked clinical worsening

**Figure 3:**
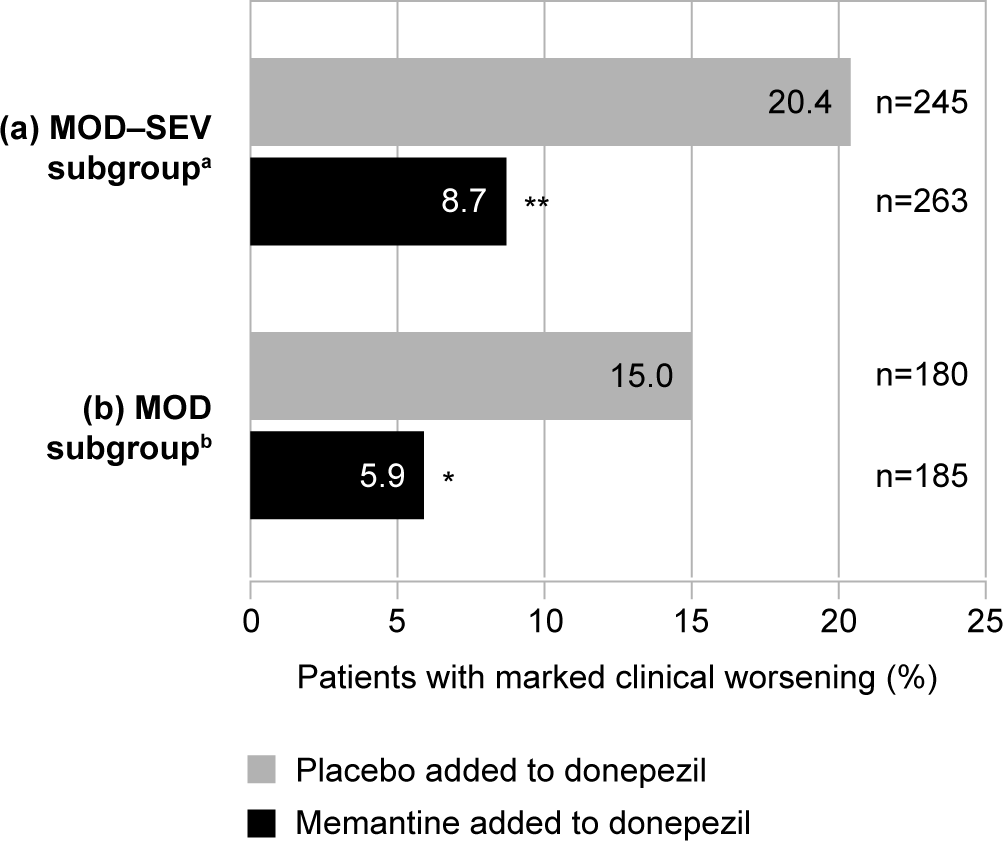
**Proportion of patients showing marked clinical worsening (ITT set, LOCF analysis)**. ^a^Moderate to severe AD (MMSE 5 to 19 at baseline), receiving donepezil (10 mg/day). ^b^Moderate AD (MMSE 10 to 19 at baseline), receiving donepezil (10 mg/day). *P < 0.01 versus placebo added to donepezil; **P < 0.001 versus placebo added to donepezil. AD, Alzheimer's disease; ITT, intention-to-treat; LOCF, last observation carried forward.

In the MOD-SEV subgroup, 23/263 patients receiving memantine added to donepezil (8.7%) showed marked clinical worsening compared to 50/245 patients receiving placebo added to donepezil (20.4%), a significant difference of 11.7% (*P *= 0.0002; LOCF) (Figure 3a). The OC analysis produced a similar result (8.5% versus 18.9%; *P *= 0.003).

In the MOD subgroup, 11/185 patients receiving memantine added to donepezil (5.9%) showed marked clinical worsening compared to 27/180 patients receiving placebo added to donepezil (15.0%), a significant difference of 9.1% (*P *= 0.006; LOCF) (Figure 3b). Again, the OC analysis produced a similar result (5.9% versus 12.6%; *P *= 0.047).

### Safety and tolerability - incidence of adverse events

The incidence of AEs over 24 weeks was similar between the patients treated with memantine added to donepezil versus placebo added to donepezil (Table 3). In the MOD-SEV subgroup, the most common AEs with an incidence ≥ 5% in patients treated with memantine added to donepezil were: dizziness, agitation, confusional state, diarrhoea, and nasopharyngitis (Table 3). In the MOD subgroup, the most common AEs with an incidence ≥ 5% in patients treated with memantine added to donepezil were: dizziness, diarrhoea, falls, and urinary tract infection (Table 3). In both severity subgroups, the frequency of agitation was statistically significantly lower in patients treated with memantine added to donepezil compared with patients treated with placebo added to donepezil (Table 3). There were no other statistically significant differences between treatment groups for AEs with an incidence ≥ 5%.

**Table 3:** Adverse events with an incidence ≥ 5% in either treatment group over 24 weeks (APT set)

	MOD-SEV subgroup^a^		MOD subgroup^b^	
	
Adverse event	Memantine added to donepezil (*n *= 269)	Placebo added to donepezil (*n *= 251)	Memantine added to donepezil (*n *= 190)	Placebo added to donepezil (*n *= 185)
Patients with AEs	206 (76.6)	186 (74.1)	144 (75.8)	136 (73.5)
Dizziness	20 (7.4)	19 (7.6)	17 (8.9)	16 (8.6)
Agitation	17 (6.3)*	29 (11.6)	9 (4.7)*	19 (10.3)
Confusional state	15 (5.6)	6 (2.4)	-	-
Diarrhoea	14 (5.2)	21 (8.4)	12 (6.3)	14 (7.6)
Nasopharyngitis	14 (5.2)	6 (2.4)	-	-
Falls	11 (4.1)	15 (6.0)	10 (5.3)	11 (5.9)
Urinary tract infection	-	-	10 (5.3)	8 (4.3)
Depression	-	-	6 (3.2)	11 (5.9)

Data are number (%). ^a^Moderate to severe AD (MMSE 5 to 19 at baseline), receiving donepezil (10 mg/day). ^b^Moderate AD (MMSE 10 to 19 at baseline), receiving donepezil (10 mg/day). *P < 0.05 versus placebo added to donepezil. APT, all-patients-treated; AE, adverse event; '-' denotes AEs with an incidence < 5% in both treatment groups in the respective severity subgroup.

## Discussion

### Efficacy

This study combined efficacy data for 510 patients with AD and MMSE < 20 from the two 24-week, phase III RCTs of memantine 20 mg/day added to stable donepezil (MEM-MD-02 and MEM-MD-12). It yielded SMD effect sizes in favour of combination treatment with memantine added to stable donepezil (versus placebo added to stable donepezil) that were in the clinically significant 0.2 to 0.4 range for all efficacy domains (Figure 2).

Study MEM-MD-02, which observed significant benefits for memantine over placebo in cognition, function, and global status, considered generally the same population of patients as the present study (MMSE 5 to 14, receiving stable donepezil therapy) [16,28,29]. Consequently, the data from MEM-MD-02 contributed favourably to the results of this meta-analysis. Regarding study MEM-MD-12 [17], only the data for the patients with moderate AD and who were taking donepezil 10 mg/day were included. In this particular subpopulation, benefits in cognition, function and global status of similar magnitude to those observed for patients in MEM-MD-02 were observed; these data demonstrated statistical significance when combined in this meta-analysis with data from the MEM-MD-02 study. These results support the hypothesis that low power and heterogeneities due to baseline ChEI treatment and disease severity may have significantly contributed to the apparently divergent results previously observed between the studies MEM-MD-02 and MEM-MD-12; these differences may have obscured the significant benefits of adding memantine to stable donepezil treatment in patients with moderate AD.

The results observed in these meta-analyses are comparable to those reported in previous meta-analyses for the benefits of memantine monotherapy over placebo [30,31]. The overall standardised effect sizes in the cognitive domain are 0.36 in the present analysis (MOD-SEV subgroup; LOCF), 0.26 in Winblad *et al. *(OC) [30] and 0.29 in Doody *et al. *(LOCF) [31]. Results found here are also comparable to previous findings in the domains of function and global status [30,31]. A major difference between this study and those previously reported is that patients in the present analysis were already receiving symptomatic benefits from donepezil, and thus the benefits observed for memantine treatment here are over and above those produced by donepezil alone. Also notable is that LOCF analyses yielded statistical significance for all domain measures (cognition, function, global status), analyses (efficacy in individual domains, marked clinical worsening), and groups/subgroups (MOD-SEV, MOD) (eight out of eight LOCF comparisons favoured combination therapy at *P *< 0.05), whereas OC analyses yielded statistical significance (*P *< 0.05) favouring combination therapy in six of eight analyses (the exceptions being cognition and function in the MOD subgroup). Nonetheless, the effect size estimates for the magnitude of clinical effects using LOCF and OC analyses were similar, and favoured combination therapy in each case. Taken together with the observation that the MOD subgroup contains only approximately two-thirds the sample size of the MOD-SEV group, and would therefore be more sensitive to the adverse effects of reductions in power from an OC analysis (compared to an LOCF analysis), these observations further support the hypothesis that lack of power, along with differences in baseline severity and ChEI type, substantially contributed to the absence of significant findings in the original MEM-MD-12 study.

While longer-term observational clinical cohort studies also support the clinical effectiveness of combination therapy above and beyond stable ChEI monotherapy [32-34], the recently published 52-week, randomised, placebo-controlled DOMINO-AD study in patients with moderate to severe AD (mean baseline standardised MMSE = 9.1), receiving stable donepezil therapy for at least 6 weeks, and whose clinician was considering a change in drug treatment, did not report similar effects [35]. This was an important study that had substantial methodological limitations, which may have particularly affected reliability and validity of results regarding detecting potential group differences over the full course of the 52-week study; these limitations included a study re-design due to delayed and insufficient recruitment, subsequently not meeting re-adjusted sample size requirements, high and imbalanced attrition causing non-ignorable missing data, and reporting of mixed effects modelling results based on difference testing, as opposed to equivalence testing, performed without a posteriori power analysis (when required sample sizes were not achieved). Overall, these limitations may have substantially biased towards null results and resulted in inadequate power to demonstrate significant differences between groups, particularly between donepezil monotherapy and memantine added to donepezil. Yet, despite these limitations, the overall results provide further evidence to support the benefits of donepezil continuation, and of memantine treatment despite discontinuation of donepezil. Though controversial and open to interpretation, potential signals of efficacy for the addition of memantine to donepezil therapy, as reflected in the initial 30 weeks of the study (during which the patient numbers were much higher compared to week 52 assessment), and in the behavioural domain, have been postulated by other researchers, or reported in the study, and warrant further secondary analysis [35,36]. Additionally, while scientific, methodological and practical considerations preclude direct comparison or inclusion of DOMINO-AD data in the current study, which was designed to assess a different hypothesis, this would be of great future interest in a suitably designed study that would broaden generalisability of findings to a wider and more heterogeneous patient population across two continents.

Long-term observational clinical cohort studies performed in naturalistic settings with prospectively collected data show similar patterns to RCTs, and demonstrate Level II grade, generalisable evidence that favours combination treatment over monotherapy, and monotherapy over placebo/no anti-dementia medication treatment [32-34]. Long-term combination therapy with memantine added to a ChEI has, in the clinical setting, been observed to significantly reduce cognitive and functional decline, and to delay time to nursing home admission compared to ChEI monotherapy and to standard care without a ChEI or memantine [32,33]. Furthermore, the benefits of combination therapy increase with time on treatment, and are sustained for years [32]. The latter observation is further supported by Rountree and colleagues who found that benefits of treatment with a ChEI and/or memantine significantly increased with treatment persistence and were observable across multiple symptom domains and stages of disease, including moderate and severe AD [37]. Finally, the recent REAL.FR cohort study, which followed 686 patients with mild to moderate AD in 16 specialised memory clinics in France (89% used ChEI monotherapy at baseline, 26% used ChEI and memantine combination therapy by year 4), reported significantly less decline in this cohort over 4 years compared to untreated historical cohorts [38].

### Clinical worsening

In the MOD-SEV subgroup, the occurrence of marked clinical worsening in patients receiving memantine added to donepezil was less than half that of those receiving placebo added to donepezil (8.5% versus 18.9%; *P *= 0.003; OC; MMSE 5 to 19). This rate is similar to the rate reported in patients receiving any concurrent ChEI (donepezil, galantamine, or rivastigmine) previously reported in a pooled clinical worsening analysis using data from the same two studies (9.8% versus 18.3%; *P *< 0.01; OC; MMSE 5 to 19) [6]. In the present study, the occurrence of marked clinical worsening in the MOD subgroup was also observed to be less than half for those treated with memantine added to donepezil versus placebo added to donepezil.

Previous reports have considered the occurrence of clinical worsening in memantine and donepezil monotherapy studies [6,13]. In data pooled from four memantine monotherapy studies, a significantly lower occurrence of marked clinical worsening was observed for memantine versus placebo (11.4% versus 23.0%; OC; week 24/28; *P *< 0.001; MMSE < 20) [6]. In data pooled from three donepezil monotherapy studies, a significantly lower occurrence of any worsening (any concurrent decline in cognition, function, and global status) was observed for donepezil versus placebo (14.4% versus 30.9%; OC; week 24; *P *< 0.0001; MMSE 10 to 17) [13].

Though improvement is most desirable, in practice, patients and caregivers accept that no worsening is also an acceptable outcome [1]. Patients with AD benefit from stabilised symptoms, as they are able to remain at a higher functional level for longer [1]. Caregivers also benefit from reduced patient decline as the patient's retained independence reduces the burden placed upon the caregiver. In the present study, since patients in the placebo group were already receiving stable donepezil treatment, the addition of memantine offered extra benefits by further reducing the occurrence of marked clinical worsening, not just in later stages, but also in moderate AD.

### Safety and tolerability

In moderate to severe AD, and moderate AD, combination treatment with memantine and donepezil was well-tolerated and had a similar incidence of AEs as treatment with placebo added to donepezil. Individually, studies MEM-MD-02 and MEM-MD-12 indicated that combination therapy with memantine added to donepezil/ChEI was safe and well-tolerated [16,17], a pattern of safety that was also recently reported in the DOMINO-AD study [35]. In the present study, the frequency of agitation was approximately half in the memantine-treated group compared with the placebo-treated group. A significant reduction in the incidence of agitation in favour of memantine monotherapy over placebo has been previously observed in a meta-analysis of patients with AD [31]. Furthermore, in a pooled analysis of patients with moderately severe to severe AD (MMSE 3 to 14) who had baseline symptoms of agitation/aggression or psychosis, a significantly greater proportion of memantine-treated patients experienced an improvement of agitation/aggression over 6 months than patients treated with placebo [39]. AE profiles from previous studies also suggest that memantine administration may be associated with amelioration of gastrointestinal AEs typically associated with ChEI use, and that rates of diarrhoea and faecal incontinence may be reduced when memantine is added to stable donepezil treatment [16,40].

### Strengths of study

This study includes data from rigorous 24-week RCTs in the largest population of moderate to severe patients treated with the combination of memantine/placebo added to donepezil considered to date (510 participants in the ITT set). The two studies included in the meta-analyses had similar inclusion/exclusion criteria, and by further restricting the MMSE range to 5 to 19 and the allowed baseline ChEI to donepezil 10 mg/day, the subjects included produced much more homogeneous groups of patients in which potential signals of efficacy could be detected. Calculation of effect sizes in this study also allows for comparisons within and between studies and to gauge the magnitude of clinical effects/clinical significance, not just statistical significance. Measuring marked clinical deterioration by combining three efficacy scales provides a powerful tool for clinicians to determine whether the patient's condition as a whole is deteriorating; a real and clinically significant deterioration that is likely to be the result of true disease progression rather than minor or statistical fluctuations that may be observed in any single domain [6]. Finally, expected treatment-related benefits must be balanced with potential treatment-related risks to provide an informed picture of the risk-benefit calculus of the treatment paradigm to patients and caregivers; this study presents the largest safety and AE data profile specifically available for the combination of memantine added to donepezil in moderate, as well as for moderate to severe AD.

### Limitations of study

Only two 24-week phase III RCTs met inclusion criteria and could thus be included, and only a subgroup of the patients from each study was included, for the reasons discussed. Also, while the MMSE has been commonly used as a disease stage proxy to determine patient inclusion in clinical trials, it only measures cognition, which is just one of several AD symptom domains, and it does so in a very limited way. The present study used MMSE in the same manner, and is thus subject to these same limitations. However, this does allow application of these results to the same criteria and populations identified by funding groups or agencies that provide treatment guidelines for the use or reimbursement of AD medications. Furthermore, measuring marked clinical worsening as defined in this study, may not capture significant decline in all patients, as a patient may largely deteriorate in one domain and yet not be considered to have marked clinical worsening; it is therefore a conservative estimate [6]. Finally, limiting data inclusion criteria in this study to test an a priori hypothesis leverages patient homogeneity, and likely lowers the odds of variability in study measures, thereby increasing the internal validity of the results, but at the cost of potentially decreasing external validity and generalisability of results to the wider and more heterogeneous non-research AD patient population; particularly the generalisability of results to those patients who are in milder clinical stages, are treated with ChEIs other than donepezil (that is, patients treated with galantamine or rivastigmine), or are enrolled in primarily non-research clinical settings. In clinical practice, patients are also often treated for much longer than the limited 24-week duration studied in these phase III RCTs. This has been emphasised to argue that Level II grade evidence from long-term observational clinical cohort studies should be used to complement and inform clinicians and researchers in the process of therapeutic discovery and assessment, as well as in determining and monitoring the long-term risk-benefit calculus of therapies to patients and to society [34].

## Conclusions

The addition of memantine to patients' ChEI therapy when they reach the moderate stage of AD has important practical relevance. The results presented in this study provide evidence in a large RCT meta-analysis population, for the significant benefits of the addition of memantine to stable donepezil therapy in moderate, as well as moderate to severe AD. These results, along with the wealth of other clinical evidence [34,41], support and extend previous findings that combination treatment is associated with clinically significant benefits in reducing 24-week decline in cognition, function, global status, and the occurrence of marked clinical worsening. In the absence of disease-modifying therapies, retaining greater cognitive and functional abilities can produce disease-course-modifying effects that may help patients with AD remain independent for longer. Importantly, combination therapy with memantine added to donepezil demonstrates good safety and tolerability, and the observed benefits are over and above those of donepezil alone. Taken together, these results support a risk-benefit calculus that is in favour of combination therapy with memantine added to donepezil in moderate, as well as moderate to severe AD, and imply translation of clinically meaningful benefits to patients, caregivers, and society.

## Abbreviations

AD: Alzheimer's disease; ADAS-Cog: Alzheimer's Disease Assessment Scale-cognitive subscale; ADCS-ADL: Alzheimer's Disease Cooperative Study-Activities of Daily Living; ADL: activity of daily living; AE: adverse event; APT: all-patients-treated; ChEI: cholinesterase inhibitor; CIBIC-Plus: Clinician's Interview-Based Impression of Change Plus Caregiver Input; IRB: Institutional Review Board; ITT: intention-to-treat; LOCF: last observation carried forward; MMSE: Mini-Mental State Examination; MOD: moderate Alzheimer's disease; MOD-SEV: moderate to severe Alzheimer's disease; NMDA: *N*-methyl-D-aspartate; OC: observed cases; RCT: randomised, double-blind, placebo-controlled trial; SIB: Severe Impairment Battery; SMD: standardised mean difference.

## Competing interests

A Atri has no equity, shares or salary from any pharma company and is not a member of any pharma speakers' bureau. In the past 5 years, he has received honoraria for educational lectures or webcasts at scientific, medical and educational conferences, meetings, programmes or advisory boards from Forest, Harvard Medical School Continuing Education, Massachusetts General Hospital Academy of Medical Educators, Lundbeck, Merck, Merz, Novartis, and Reed-Elsevier Medical Education. Institutional research grant funding has been received from Forest for research unrelated to this study and manuscript. J Molinuevo has no equity, shares or salary from any pharma company. He has received honoraria for speaking and for attending advisory boards from Lundbeck, Merck, Merz, and Novartis. O Lemming is a full-time employee of H. Lundbeck A/S. Y Wirth is a former full-time employee of Merz Pharmaceuticals GmbH. I Pulte is a full-time employee of Merz Pharmaceuticals GmbH. D Wilkinson has no equity, shares or salary from any pharma company. He has received honoraria for speaking and for attending advisory boards from Lundbeck, Merz, Pfizer, and Nutricia. A Atri, J Molinuevo, and D Wilkinson did not receive financial support or remuneration related to work on this study or manuscript.

## Authors' contributions

OL, YW and IP performed the analyses of the data and reviewed the manuscript. AA, JM and DW provided the interpretation and discussion of the data analyses outcome. All authors contributed to manuscript preparation, and read and approved the final manuscript.

## References

[B1] Geldmacher DS Frolich L Doody RS Erkinjuntti T Vellas B Jones RW Banerjee S Lin P Sano M Realistic expectations for treatment success in Alzheimer's disease J Nutr Health Aging 2006 10 417 429 17066215

[B2] Cummings JL Challenges to demonstrating disease-modifying effects in Alzheimer's disease clinical trials Alzheimers Dement 2006 2 263 271 10.1016/j.jalz.2006.07.001 19595897 PMC5854177

[B3] Folstein MF Folstein SE McHugh PR "Mini-mental state". A practical method for grading the cognitive state of patients for the clinician J Psychiat Res 1975 12 189 198 10.1016/0022-3956(75)90026-6 1202204

[B4] Winblad B Poritis N Memantine in severe dementia: results of the M-Best Study (Benefit and efficacy in severely demented patients during treatment with memantine) Int J Geriatr Psychiatry 1999 14 135 146 10.1002/(SICI)1099-1166(199902)14:2<135::AID-GPS906>3.0.CO;2-0 10885864

[B5] Reisberg B Doody R Stöffler A Schmitt F Ferris S Möbius HJ Memantine Study Group Memantine in moderate-to-severe Alzheimer's disease N Engl J Med 2003 348 1333 1341 10.1056/NEJMoa013128 12672860

[B6] Wilkinson D Andersen HF Analysis of the effect of memantine in reducing the worsening of clinical symptoms in patients with moderate to severe Alzheimer's disease Dement Geriatr Cogn Disord 2007 24 138 145 10.1159/000105162 17622761

[B7] Rogers SL Farlow MR Doody RS Mohs R Friedhoff LT A 24-week, double-blind, placebo-controlled trial of donepezil in patients with Alzheimer's disease. Donepezil Study Group Neurology 1998 50 136 145 10.1212/WNL.50.1.136 9443470

[B8] Feldman H Gauthier S Hecker J Vellas B Subbiah P Whalen E Donepezil MSAD Study Investigators Group A 24-week, randomized, double-blind study of donepezil in moderate to severe Alzheimer's disease Neurology 2001 57 613 620 10.1212/WNL.57.4.613 11524468

[B9] Winblad B Engedal K Soininen H Verhey F Waldemar G Wimo A Wetterholm AL Zhang R Haglund A Subbiah P Donepezil Nordic Study Group A 1-year, randomized, placebo-controlled study of donepezil in patients with mild to moderate AD Neurology 2001 57 489 495 10.1212/WNL.57.3.489 11502918

[B10] Winblad B Black SE Homma A Schwam EM Moline M Xu Y Perdomo CA Swartz J Albert K Donepezil treatment in severe Alzheimer's disease: a pooled analysis of three clinical trials Curr Med Res Opin 2009 25 2577 2587 19735164 10.1185/03007990903236731

[B11] Birks J Harvey RJ Donepezil for dementia due to Alzheimer's disease Cochrane Database Syst Rev 2006 CD001190 10.1002/14651858.CD001190.pub2 16437430

[B12] McShane R Areosa Sastre A Minakaran N Memantine for dementia Cochrane Database Syst Rev 2006 CD003154 10.1002/14651858.CD003154.pub5 16625572

[B13] Wilkinson D Schindler R Schwam E Waldemar G Jones RW Gauthier S Lopez OL Cummings J Xu Y Feldman HH Effectiveness of donepezil in reducing clinical worsening in patients with mild-to-moderate Alzheimer's disease Dement Geriatr Cogn Disord 2009 28 244 251 10.1159/000241877 19786776 PMC3202931

[B14] Gauthier S Molinuevo JL Benefits of combined cholinesterase inhibitor and memantine treatment in moderate-severe Alzheimer's disease Alzheimers Dement 2012 published online 27 October 2012, doi:10.1016/j.jalz.2011.11.005 10.1016/j.jalz.2011.11.005 23110864

[B15] Periclou AP Ventura D Sherman T Rao N Abramowitz WT Lack of pharmacokinetic or pharmacodynamic interaction between memantine and donepezil Ann Pharmacother 2004 38 1389 1394 10.1345/aph.1D638 15266045

[B16] Tariot PN Farlow MR Grossberg GT Graham SM McDonald S Gergel I Memantine Study Group Memantine treatment in patients with moderate to severe Alzheimer disease already receiving donepezil: a randomized controlled trial JAMA 2004 291 317 324 10.1001/jama.291.3.317 14734594

[B17] Porsteinsson AP Grossberg GT Mintzer J Olin JT Memantine MEM-MD-12 Study Group Memantine treatment in patients with mild to moderate Alzheimer's disease already receiving a cholinesterase inhibitor: a randomized, double-blind, placebo-controlled trial Curr Alzheimer Res 2008 5 83 89 10.2174/156720508783884576 18288936

[B18] Saxton J McGonigle KL Swihart AA Boller F The Severe Impairment Battery 1993 Bury St Edmunds: Thames Valley Test Company

[B19] Panisset M Roudier M Saxton J Boller F Severe impairment battery. A neuropsychological test for severely demented patients Arch Neurol 1994 51 41 45 10.1001/archneur.1994.00540130067012 8274108

[B20] Schmitt FA Ashford W Ernesto C Saxton J Schneider LS Clark CM Ferris SH Mackell JA Schafer K Thal LJ The severe impairment battery: concurrent validity and the assessment of longitudinal change in Alzheimer's disease. The Alzheimer's Disease Cooperative Study Alzheimer Dis Assoc Disord 1997 11 Suppl 2 51 56 9236953

[B21] Rosen WG Mohs RC Davis KL A new rating scale for Alzheimer's disease Am J Psychiatry 1984 141 1356 1364 6496779 10.1176/ajp.141.11.1356

[B22] Galasko D Bennett D Sano M Ernesto C Thomas R Grundman M Ferris S An inventory to assess activities of daily living for clinical trials in Alzheimer's disease. The Alzheimer's Disease Cooperative Study Alzheimer Dis Assoc Disord 1997 11 Suppl 2 33 39 9236950

[B23] Galasko D Schmitt F Thomas R Jin S Bennett D Alzheimer's Disease Cooperative Study Detailed assessment of activities of daily living in moderate to severe Alzheimer's disease J Int Neuropsychol Soc 2005 11 446 453 16209425 10.1017/s1355617705050502

[B24] Reisberg B Schneider L Doody R Anand R Feldman H Haraguchi H Kumar R Lucca U Mangone CA Mohr E Morris JC Rogers S Sawada T Clinical global measures of dementia. Position paper from the International Working Group on Harmonization of Dementia Drug Guidelines Alzheimer Dis Assoc Disord 1997 11 Suppl 3 8 18 9305508

[B25] Schneider LS Olin JT Doody RS Clark CM Morris JC Reisberg B Schmitt FA Grundman M Thomas RG Ferris SH Validity and reliability of the Alzheimer's Disease Cooperative Study-Clinical Global Impression of Change. The Alzheimer's Disease Cooperative Study Alzheimer Dis Assoc Disord 1997 11 Suppl 2 22 32 9236949 10.1097/00002093-199700112-00004

[B26] Cohen J Statistical Power Analysis for the Behavioral Sciences 1988 Second New York, NY: Psychology Press, Taylor & Francis Group

[B27] Rockwood K Size of the treatment effect on cognition of cholinesterase inhibition in Alzheimer's disease J Neurol Neurosurg Psychiatry 2004 75 677 685 10.1136/jnnp.2003.029074 15090558 PMC1763555

[B28] Schmitt FA van Dyck CH Wichems CH Olin JT Memantine MEM-MD-02 Study Group Cognitive response to memantine in moderate to severe Alzheimer disease patients already receiving donepezil: an exploratory reanalysis Alzheimer Dis Assoc Disord 2006 20 255 262 10.1097/01.wad.0000213860.35355.d4 17132970

[B29] Feldman HH Schmitt FA Olin JT Activities of daily living in moderate-to-severe Alzheimer disease: an analysis of the treatment effects of memantine in patients receiving stable donepezil treatment Alzheimer Dis Assoc Disord 2006 20 263 268 10.1097/01.wad.0000213859.35355.59 17132971

[B30] Winblad B Jones RW Wirth Y Stöffler A Möbius HJ Memantine in moderate to severe Alzheimer's disease: a meta-analysis of randomised clinical trials Dement Geriatr Cogn Disord 2007 24 20 27 10.1159/000102568 17496417

[B31] Doody RS Tariot PN Pfeiffer E Olin JT Graham SM Meta-analysis of six-month memantine trials in Alzheimer's disease Alzheimers Dement 2007 3 7 17 10.1016/j.jalz.2006.10.004 19595910

[B32] Atri A Shaughnessy LW Locascio JJ Growdon JH Long-term course and effectiveness of combination therapy in Alzheimer disease Alzheimer Dis Assoc Disord 2008 22 209 221 10.1097/WAD.0b013e31816653bc 18580597 PMC2718545

[B33] Lopez OL Becker JT Wahed AS Saxton J Sweet RA Wolk DA Klunk W Dekosky ST Long-term effects of the concomitant use of memantine with cholinesterase inhibition in Alzheimer disease J Neurol Neurosurg Psychiatry 2009 80 600 607 10.1136/jnnp.2008.158964 19204022 PMC2823571

[B34] Atri A Rountree SD Lopez OL Doody RS Validity, significance, strengths, limitations, and evidentiary value of real-world clinical data for combination therapy in Alzheimer's disease: comparison of efficacy and effectiveness studies Neurodegener Dis 2012 10 170 174 10.1159/000335156 22327239 PMC3702018

[B35] Howard R McShane R Lindesay J Ritchie C Baldwin A Barber R Burns A Dening T Findlay D Holmes C Hughes A Jacoby R Jones R Jones R McKeith I Macharouthu A O'Brien J Passmore P Sheehan B Juszczak E Katona C Hills R Knapp M Ballard C Brown R Banerjee S Onions C Griffin M Adams J Gray R Donepezil and memantine for moderate-to-severe Alzheimer's disease N Engl J Med 2012 366 893 903 10.1056/NEJMoa1106668 22397651

[B36] Shaw G Two drugs are not better than one for treating moderate to severe Alzheimer disease, British investigators report: why some US neurologists don't agree Neurology Today 2012 12 12 13

[B37] Rountree SD Chan W Pavlik VN Darby EJ Siddiqui S Doody RS Persistent treatment with cholinesterase inhibitors and/or memantine slows clinical progression of Alzheimer disease Alzheimers Res Ther 2009 1 7 10.1186/alzrt7 19845950 PMC2874259

[B38] Gillette-Guyonnet S Andrieu S Nourhashemi F Gardette V Coley N Cantet C Gauthier S Ousset PJ Vellas B REAL.FR study group Long-term progression of Alzheimer's disease in patients under antidementia drugs Alzheimers Dement 2011 7 579 592 10.1016/j.jalz.2011.02.009 22055975

[B39] Wilcock GK Ballard CG Cooper JA Loft H Memantine for agitation/aggression and psychosis in moderately severe to severe Alzheimer's disease: a pooled analysis of 3 studies J Clin Psychiatry 2008 69 341 348 10.4088/JCP.v69n0302 18294023

[B40] Olin JT Bhatnagar V Reyes P Koumaras B Meng X Brannan S Safety and tolerability of rivastigmine capsule with memantine in patients with probable Alzheimer's disease: a 26-week, open-label, prospective trial (Study ENA713B US32) Int J Geriatr Psychiatry 2010 25 419 426 10.1002/gps.2355 19670390

[B41] Patel L Grossberg GT Combination therapy for Alzheimer's disease Drugs Aging 2011 28 539 546 10.2165/11591860-000000000-00000 21721598

